# *Abelmoschus manihot* – a traditional Chinese medicine versus losartan potassium for treating IgA nephropathy: study protocol for a randomized controlled trial

**DOI:** 10.1186/s13063-016-1774-6

**Published:** 2017-04-11

**Authors:** Ping Li, Yi-zhi Chen, Hong-li Lin, Zhao-hui Ni, Yong-li Zhan, Rong Wang, Hong-tao Yang, Jing-ai Fang, Nian-song Wang, Wen-ge Li, Xue-feng Sun, Xiang-mei Chen

**Affiliations:** 1 Department of Nephrology, State Key Laboratory of Kidney Disease, 2011DAV00088, Chinese PLA General Hospital, Beijing, 100853 China; 2 Department of Nephrology, The First Affiliated Hospital of Dalian Medical University, Dalian, Liaoning 116011 China; 3 Department of Nephrology, Renji Hospital, Shanghai Jiao Tong University School of Medicine, Shanghai, 200127 China; 4 Department of Nephrology, Guang’ anmen Hospital, China Academy of Chinese Medical Sciences, Beijing, 100053 China; 5 Department of Nephrology, Shandong Provincial Hospital, Jinan, 250021 China; 6 Department of Nephrology, First Teaching Hospital of Tianjin University of TCM, Tianjin, 300192 China; 7 Department of Nephrology, The First Hospital of Shanxi Medical University, Taiyuan, 030024 China; 8 Department of Nephrology, The Sixth Affiliated Hospital of Shanghai Jiao Tong University School of Medicine, Shanghai, 200233 China; 9 Department of Nephrology, China-Japan Friendship Hospital, Beijing, 100029 China; 10 Department of Nephrology, Chinese PLA General Hospital, Chinese PLA Institute of Nephrology, State Key Laboratory of Kidney Diseases, National Clinical Research Center for Kidney Diseases, Beijing Key Laboratory of Kidney Diseases, Fuxing Road 28, Beijing, 100853 People’s Republic of China

**Keywords:** IgA nephropathy, Traditional Chinese medicine, *Abelmoschus manihot*, Randomized controlled study

## Abstract

**Background:**

IgA nephropathy (IgAN) is one of the most common primary glomerular diseases worldwide, but effective therapy remains limited and many patients progress to end-stage renal disease (ESRD). Only angiotensin-converting enzyme inhibitors (ACE-I)/angiotensin-receptor blockers (ARB) show a high level of evidence (1B level) of being of value in the treatment for IgAN according to the 2012 Kidney Disease: Improving Global Outcomes (KDIGO) guidelines. However, traditional Chinese medicine has raised attention in kidney disease research. *Abelmoschus manihot*, a single medicament of traditional Chinese medicine has shown therapeutic effects in primary glomerular disease according to the randomized controlled clinical trial that we have completed. Here, we conduct a new study to assess the efficacy and safety of *Abelmoschus manihot* in IgAN. Also, this study is currently the largest double-blind, randomized controlled registered clinical research for the treatment of IgAN.

**Methods:**

We will conduct a multicenter, prospective, double-blind, double-dummy randomized controlled study. The study is designed as a noninferiority clinical trial. Approximately 1600 biopsy-proven IgAN patients will be enrolled at 100 centers in China and followed up for as long as 48 weeks. IgAN patients will be randomized assigned to the *Abelmoschus manihot* group (in the form of a *huangkui* capsule, 2.5 g, three times per day) and the losartan potassium group (losartan potassium, 100 mg/d). The primary outcome is the change in 24-h proteinuria from baseline after 48 weeks of treatment. Change in estimated glomerular filtration rate (eGFR) from baseline after 48 weeks of treatment, the incidence of endpoint events (proteinuria ≥3.5 g/24 h, the doubling of serum creatinine, or receiving blood purification treatment) are the secondary outcomes. Twenty-four-hour proteinuria and eGFR are measured at 0, 4, 12, 24, 36 and 48 weeks.

**Discussion:**

This study will be of sufficient size and scope to evaluate the efficacy and safety of *Abelmoschus manihot* compared to losartan potassium in treating patients with IgAN. The results of this study may provide a new, effective and safe treatment strategy for IgAN.

**Trial registration:**

ClinicalTrials.gov, identifier: NCT02231125. Registered on 30 August 2014.

**Electronic supplementary material:**

The online version of this article (doi:10.1186/s13063-016-1774-6) contains supplementary material, which is available to authorized users.

## Background

IgA nephropathy (IgAN) is the most common primary glomerular disease worldwide, and it is an important cause of chronic kidney disease (CKD) and end-stage renal disease (ESRD) [1, 2]. IgA nephropathy is also the most common primary glomerular disease and the leading cause of ESRD in China [3]. Kidney Disease: Improving Global Outcomes (KDIGO) Clinical Practice Guideline for Glomerulonephritis recommends angiotensin-converting enzyme inhibitors (ACE-I)/angiotensin-receptor blocker (ARB), corticosteroids, and immunosuppressive agent treatment according to the level of the urinary protein and renal function of IgAN patients [4]. Since current therapy of IgAN is limited and most treatments have little evidence to recommend them, it is necessary to explore novel, effective and safe treatment for IgAN patients. Traditional Chinese medicine (TCM) has a long history in treatment of chronic kidney disease (CKD), and a growing number of studies have confirmed that TCM has the curative effect of reducing urinary protein, protecting kidney function [5]. *Abelmoschus manihot* (AM) extracted from *Flos Abelmoschus manihot*, a single medicament of TCM has been widely used to treat chronic kidney diseases, such as IgAN, membranous nephropathy and diabetic nephropathy, and has shown the effects of reducing proteinuria and protecting kidney function [6–8]. ACE-I and ARB drugs are commonly used to treat proteinuria, and the range of reduction is reportedly 30–50% in adults [9, 10]. Thus, we use losartan as the positive control. We have finished a randomized controlled trial (RCT) to assess the efficacy and safety of AM in patients with primary glomerular disease. The clinical research enrolled a total of 417 patients from 26 hospitals who had been diagnosed with primary glomerular disease by renal biopsy. The results showed that AM can significantly reduce urinary protein in patients with primary kidney disease (CKD stages 1–2) and its effect is better than that of losartan potassium (50 mg/d) [11]. Since IgAN was the most common primary glomerular disease and the design of the former study had some limitations, such as not using a blind method, the low dose of losartan potassium (50 mg/d) used, and a short observation time (24 weeks), we conducted this prospective, double-blind, double-dummy randomized controlled study to evaluate efficacy and safety of AM in the treatment of IgAN.

## Methods

### Objectives

The objective of the trial is to assess the clinical effects and safety of AM in IgAN patients.

### Trial design

**Fig. 1:**
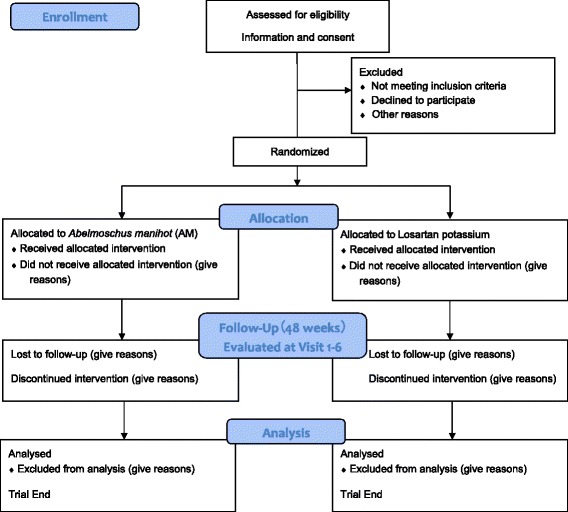
Trial flow diagram

This study is a multicenter, prospective, double-blind, double-dummy RCT. The study is designed as a noninferiority clinical trial. The hypothesis is that AM significantly reduces the urinary protein levels of IgAN patients and that its curative effect is not inferior to that of losartan potassium (100 mg/d) after 48 weeks of treatment. We have followed the Standardised Protocol Interventions: Recommendations for Interventional Trials (SPIRIT) 2013 Statement which defines standard protocol items for clinical trials [12]. See the items in Fig. 1 and Additional file 1 for the Checklist. The study protocol was designed by members of the Executive Committee (composed of the Department of Kidney Disease of Chinese People’s Liberation Army General Hospital, Peking University Clinical Research Institute). The protocol is being implemented by the study group comprising 100 clinical centers across China. The network database was designed by the Executive Committee. Venturepharm CRO Service Group which provides the clinical research associate service.

### Setting and participants

Approximately 1600 biopsy-proven IgAN patients will be enrolled at 100 centers in China and followed up for as long as 48 weeks.

### Eligibility, exclusion and exit criteria

The inclusion criteria are: (1) diagnosis of IgAN by kidney biopsy, (2) age 18 to 65 years, (3) blood pressure of ≤140/90 mmHg, (4) estimated glomerular filtration rate (eGFR) ≥45 ml/min/1.73 m^2^ (calculated with the use of the CKD-EPI Creatinine Equation 2009) [13], (5) 24-h proteinuria range of between 0.5 and 3.0 g, (6) agreement to participate and provision of signed informed consent. Additional file 2 shows the Informed Consent Form in more detail. The exclusion criteria are: (1) secondary IgAN, (2) allergy to AM or losartan potassium, (3) history of AM and ACE-I and/or ARB use within the last 6 months, (4) history of glucocorticoid, immunosuppressant, or *Tripterygium* drug use within the previous 1 year, (5) blood pressure of <90/60 mmHg, (6) serum potassium >5.5 mmol/L, (7) serum albumin <30 g/L, (8) unilateral or bilateral renal artery stenosis, (9) pregnant or lactating women, (10) severe heart, brain, liver, or hematopoietic system disease, or other serious illnesses that may affect survival, (11) participation in another clinical investigation. The exit criteria are: (1) endpoint event: proteinuria ≥3.5 g/24 h, doubling of serum creatinine, or receiving blood purification treatment, (2) blood pressure of >160/90 mmHg after antihypertensive drug combination therapy (convinced by measuring blood pressure three times within 2 weeks), (3) blood pressure of <90/60 mmHg after stopping using other antihypertensive agents, (4) serum potassium >5.5 mmol/L after antihyperkalemia drug treatment, (5) serum creatinine rapidly rising to >50% of the baseline within 4 weeks after treatment, (6) serious adverse events (SAEs): hospitalization (initial or prolonged), disability or permanent damage, life-threatening, death, congenital anomaly/birth defect and other serious events (important medical events) [14], (7) serious breach of protocol: participants failing to take medications according to protocol or taking some drugs that have a significant impact on the primary and secondary outcomes during the 48-week observation period, (8) loss to follow-up or withdrawal from the trial, (9) pregnancy during the trial.

### Interventions

*Abelmoschus manihot* (AM) group: a *huangkui* capsule given orally, 2.5 g three times per day after meals, and a losartan potassium tablet dummy 100 mg/day in the morning.

Losartan group: a losartan potassium tablet given orally, 100 mg/day in the morning and a *huangkui* capsule dummy at 2.5 g three times per day after meals.

*Abelmoschus manihot* (AM): *huanghui* capsule (Jiangsu Suzhong Pharmaceutical Group Co., Ltd.), 0.5 g × 30 capsules/box. A *huangkui* capsule is a single, plant drug extract of *Flos Abelmoschus manihot*. The main composition is of flavonoids. Content: dry extract (powder), 80%; magnesium stearate, 3%; and calcium hydrogen phosphate, 17%. The medicinal parts are the corolla with the stamens and the style. The plant is picked in early August to late October (flowering period), undergoes alcohol extraction into ambrette fluid extract, and is then vacuum-dried and crushed into a dry-extract powder. Pharmaceutical preparation does not involve boiling. The *huanghui* capsule was approved by the China Food and Drug Administration in 1999 (Z19990040, approval date: 13 August 1999).

Losartan potassium (Hangzhou MSD Pharmaceutical Co., Ltd.), 100 mg × 7 capsules/box.

The *Huanghui* capsule dummy and the losartan potassium dummy are produced by Jiangsu Suzhong Pharmaceutical Group Co., Ltd.

Treatment continues for 48 weeks. Patients whose blood pressures are >140/90 mmHg after treatment will be given calcium channel blockers. Appropriate treatment is also given when patients develop hyperlipidemia, infection, or a hypercoagulable state. Glucocorticoids and immunosuppressive agents are prohibited. To improve adherence to intervention protocols, patients will be asked to take back the drug kit at every visit to exchange the new drug and whether the drug has been taken will be recorded.

### Study outcomes

#### Primary and secondary outcome measure

The primary outcome is the change in 24-h proteinuria from baseline after 48 weeks of treatment. For this measurement, patients are instructed to collect urine over 24 h (from 07:00 to 07:00 the next day). Urine output and the total amount are measured and recorded. Twenty-four-hour urinary protein excretion is calculated based on the concentration and 24-h urine volume. The secondary outcome measures are the change in eGFR from baseline after 48 weeks treatment and the incidents of endpoint events (proteinuria ≥3.5 g/24 h, doubling of serum creatinine, or receiving blood purification treatment). All the urine and blood samples are delivered to the central laboratory within 4 weeks for testing.

#### Other measurements

The patients’ general condition, demographic data, clinical history, physical examination, blood pressure and renal biopsy report are recorded. Laboratory assessments include routine urine test, routine blood test, blood biochemical tests (alanine aminotransferase, aspartate aminotransferase, triglyceride, cholesterol, low-density lipoprotein level, total bilirubin, direct bilirubin, gamma glutamyl transpeptidase, total protein, albumin, serum creatinine, blood urea nitrogen, uric acid, blood glucose and potassium). Enrolled patients also undergo electrocardiogram and chest X-ray examinations. Central laboratory tests include 24-h urinary proteinuria, urine routine test, serum cystatin C and high-sensitivity C-reactive protein. The urine and blood samples are both stored in a −80 °C refrigerator at central laboratory and participating centers.

### Safety

Safety evaluation includes the patients’ general condition, the incidence of adverse events, and laboratory assessments. Adverse events and SAEs are defined according to the definitions of Good Clinical Practice (GCP) by the China Food and Drug Administration (CFDA) [14].

### Participant follow-up

The enrollment process includes a screening visit to assess eligibility and obtain the informed consent and collection of clinical and demographic information. After enrollment, participants are evaluated at visit 1 (baseline visit), visit 2 (4 weeks), visit 3 (12 weeks), visit 4 (24 weeks) and visit 5 (36 weeks) and visit 6 (48 weeks) with similar data collection as performed in the baseline visit (Table 1).

**Table 1:** Study visits

Item	Screening visit	V1	V2	V3	V4	V5	V6
Visit							
Time	−1w	0w ± 7d	4w ± 7d	12w ± 7d	24w ± 7d	36w ± 7d	48w ± 7d
Confirm eligibility	√	√					
Written informed consent	√						
Clinical and demographic information	√	√					
Physical examination	√	√	√	√	√	√	√
Signs and symptoms	√	√	√	√	√	√	√
24-h urinary proteinuria	√	√	√	√	√	√	√
24-h urine creatinine	√	√	√	√	√	√	√
Routine urine test	√	√	√	√	√	√	√
Urine pregnancy test		√					
Routine blood test		√	√	√	√	√	√
Blood biochemical tests		√	√	√	√	√	√
eGFR	√	√	√	√	√	√	√
Serum cystatin C and high-sensitivity C-reactive protein		√			√		√
Electrocardiogram and		√					√
Chest X-ray examinations		√					√
Drug distribution		√	√	√	√	√	
Drug combination		√	√	√	√	√	√
Adverse event			√	√	√	√	√
Exit criteria assessment			√	√	√	√	√

Note: the urine pregnancy test is screened in fertile women aged from 18 to 50 years

### Sample size

The sample size determination is based on the primary outcome 24-h proteinuria. The alpha level is set to a one-sided value of 0.025%. We assume that the expected decline of 24-h proteinuria is 1600 ± 500 mg. The noninferiority margin (0.5 of the estimated standard deviation) is 250 mg. With a 15% dropout rate, to enroll 124 patients per arm will offer 95% power to demonstrate noninferiority of AM compared to losartan with a 250-mg noninferiority margin. At the same time, based on the observation of safety, sample size expanded to 800 patients per arm will offer 80% power to detect the rare adverse reactions (incidence <0.5%) at least in one patient.

### Recruitment

The process of recruiting participants will be based on medical record review. All the hospitalized patients undergoing a kidney biopsy from September 2014 and the outpatients with a documented history of IgAN by kidney biopsy within 1 year will be screened according to the inclusion and exclusion criteria. There are 100 participating clinical centers and every center will need to enroll at least 12 patients.

### Randomization procedure, allocation concealment, implementation of blind method

Consecutive numbers are assigned to each participating center according to a center-stratified random order generated by SAS 9.4 Proc Plan. Peking University Clinical Research Institute takes the responsibility of generating the allocation sequence and an interactive voice-response or web-response system will be used to assign each patient a number and group. The centers then randomly assign patients to the AM or losartan potassium treatment group. The allocation ratio is 1:1. The test uses a double-dummy drug; therefore, in the whole course of the trial, the patients, the doctors and the statistical analysts of the trial are unaware of the treatments. In patients with SAEs, an emergency unblinding procedure will be initiated.

### Data collection and management

Table 1 gives an overview of assessment tests and the time of data collection. For each patient who is randomly assigned to a group, regardless of their final completion of the experiment, the baseline data and follow-up data will be saved. Patients enrolled in the trial will be treated preferentially and checked for free. The clinical investigators from each center collect the data and fill in the Case Report Forms. For keeping intact, first-hand data, we also design specified medical records for the clinical trial. Researchers write the medical records at the same time as making follow-up observations on the patients and ensure that the data records are timely, complete, accurate and true. The inspectors of the Venturepharm CRO Service Group will review the data. The inspectors are responsible for checking the consistency of all data sources, paper Case Report Forms and electronic databases. Original medical records and informed consents are archived in the participating centers and saved for at least 5 years after the clinical trials finish. The General Hospital of PLA is responsible for the establishment of network data registration platform, and investigators are responsible for the paper case report table entry to the platform. All data will be transferred to the data statistical units for data entry and management with the EpiData3.1 database.

### Statistical methods

The statistical analysis plan will be decided before database lock and unblinding of the trial. Statistical analysis for the trial will be performed by the Peking University Clinical Research Institute. Statistics software SAS 9.4 will be used for the data analysis. Data analysis follows the principles of intention-to-treat analysis (ITT). The full analysis set is defined as all randomly assigned patients with both a baseline and a post-baseline assessment. Last observation carried forward (LOCF) will be used to handle the missing data. Categorical variables are presented as counts with percentages and compared by the chi-square test or Fisher’s exact test. Continuous variables are presented as mean with standard deviation and compared by *t* test if the variables are normal distribution or Mann-Whitney *U* test if the variables are non-normally distributed. A *t* test (two independent samples) will be conducted to compare the primary and secondary outcomes. An analysis of covariance (ANCOVA) model for the primary outcome will be conducted considering baseline proteinuria and study center effect. The LS-means and 95% LS confidence intervals will be generated.

### Monitoring

#### Data monitoring

The Data Monitoring Committee (DMC) is composed of The Implement and Management Office of the Significant New Drug Creation of the National Science and Technology Major Projects and the Clinical Research Management Center of PLA General Hospital.

#### Harms

Potential adverse events include dizziness, gastrointestinal discomfort, rash, itching, amenorrhea and cough. In the process of the clinical trial, any SAEs must be immediately reported to the principal investigator, the Medical Ethics Committee of the Chinese People’s Liberation Army General Hospital, and to Safety Supervision of CFDA within 24 h. At the same time, the researchers must fill in the record of SAEs including the time that the SAEs occur, their severity and duration and the measures and outcome. Adverse events are recorded every 4 weeks from the baseline visit until the final visit. SAEs are recorded up to 30 days after the final visit.

#### Auditing

The Implement and Management Office of the Significant New Drug Creation of the National Science and Technology Major Projects will audit this trial every 6 months and it is independent from the investigators and the sponsor.

### Dissemination

During its implementation in clinical trials, if the plan need to be revised, the principle investigator is responsible for the revision of the plan and for re-submitting it to the Ethics Committee for approval after consultation and discussion with the Executive Committee. Clinicians in each study center are responsible for the patient’s consent or assent. The enrolled patients have also signed the consent for blood and urine sample collection. All patient data will be saved according to the assigned serial number and the private information will be protected. The principal investigator declares that there are no competing interests for the overall trial and each study site. After all patients complete follow-up data records, the database will be locked by Peking University Clinical Research Institute. No person will obtain the data before analysis has finished. We plan to report the trial results for publication in an appropriate journal and to communicate the results at an academic conference. The Executive Committee will write the report and our final report will follow Consolidated Standards of Reporting Trials (CONSORT) guidelines [15].

## Discussion

Due to the low quality of guideline evidence, the efficacy of drug treatment is still lack in IgAN patients. Since the 2012 KDIGO guidelines were released, the results of two multicenter, large-sample RCTs are worth looking at. The STOP-IgAN trial has not yet confirmed that glucocorticoid and other immunosuppressive agents can significantly improve the long-term prognosis of patients with IgN [16]. Another clinical trial evaluating the efficacy and safety of glucocorticoids (TESTING test) is still ongoing, and our center also participated in this study [17]. Therefore, it is imperative to find new nonimmune suppressive drugs for IgAN. The efficacy of TCM has been confirmed in some randomized controlled studies in recent years [18] and great progress has been made recently in the field of TCM, but large and well-designed RCTs are still lacking.

The flowers of *Abelmoschus manihot* (Linn.) Medicus (family Malvaceae) have been used as a traditional treatment of chronic glomerulonephritis in China for centuries and the flowers are widely distributed in China. The major biologically active constituents are flavonoids [19]. High-performance liquid chromatography (HPLC) and ultra-performance liquid chromatography (UPLC)/quadrupole time-of-flight mass spectrometry (QTOF MS) can be both used to determine the content of the main chemical constituents of AM. Therefore, they can be used for standardization of AM production [20, 21]. Recent studies have indicated that flavonoids can be converted to a combination of acid sulfates in animals, which may be the main active metabolic ingredient for kidney protection [22]. For the mechanistic studies, AM has shown to improve kidney inflammation and glomerular injury in rats with doxorubicin-induced nephropathy through inhibition of the p38 MAPK signaling pathway and TGF-β1 protein expression [23]. The results of our previous study have shown that AM significantly reduced proteinuria and maintained kidney function in patients with primary glomerular disease, non-nephrotic-range proteinuria and normal kidney function [11]. AM even showed a better therapeutic effect on reducing proteinuria than losartan (50 mg/d) after 24 weeks of treatment [24]. In addition, the combination of AM and losartan showed better efficacy than losartan monotherapy. Studies have suggested that high doses of ARBs could retard the progression of kidney disease in hypertensive and/or diabetic patients [25, 26]. This study was based on losartan at 100 mg/day as a positive control, and one of the inclusion criteria was effective control of hypertension (blood pressure of ≤140/90 mmHg); thus, we do not suggest changing the dosage of losartan. Other studies have shown that AM has good efficacy in patients with nephrotic syndrome and diabetic nephropathy [27–30]. Our team has carried out meta-analysis evaluating the efficacy and safety of the *Huangkui* capsule in the treatment of chronic kidney disease. The analysis included all RCTs and non-randomized controlled trials published between January 1995 and July 2011. The results showed that AM can reduce urinary protein in both nephrotic syndrome and nephritis syndrome [31]. Our team also conducted the meta-analysis evaluating the efficacy and safety of the *Huangkui* capsule in the treatment of diabetic nephropathy (urinary protein of 0.5–3.5 g/24 h). AM showed the effect of reducing urinary protein in diabetic nephropathy. According to the previous studies, AM is well tolerated with minimal adverse events [32].

To further evaluate the efficacy and safety of AM in the treatment of IgAN, we are conducting this study comparing AM and losartan (100 mg/d) which will enroll 1600 IgAN patients. In conclusion, the results of this trial will provide clinical evidence for the efficacy and safety of AM in the treatment of patients with stages 1 to 3 CKD with IgAN.

### Trial status

This trial began recruiting participants in October 2015. We have recruited 912 patients in 87 hospitals as of 13 June 2016. We estimate to have collected post-treatment and follow-up data by the winter of 2017.

## Additional files

Additional file 1: SPIRIT 2013 Checklist: recommended items to address in a clinical trial protocol and related documents. (DOC 123 kb)

Additional file 2: Informed Consent Form. A Multi-center, Double-blind, Double-simulation and Randomized Control Study of Huangkui Capsule for the Treatment of IgA Nephropathy: Informed Consent. (PDF 85 kb)

## Abbreviations

ACE-I: Angiotensin-converting enzyme inhibit
AM: *Abelmoschus manihot*
ARB: Angiotensin-receptor blocker
CFDA: China Food and Drug Administration
CKD: Chronic kidney disease
eGFR: Estimated glomerular filtration rate
ESRD: End-stage renal disease
GCP: Good Clinical Practice
IgAN: IgA nephropathy
ITT: Intention-to-treat analysis
KDIGO: Kidney Disease: Improving Global Outcomes
SAEs: Serious adverse events
TCM: Traditional Chinese medicine
