# Supplementary material for: Abelmoschus manihot – a traditional Chinese medicine versus losartan potassium for treating IgA nephropathy: study protocol for a randomized controlled trial
Source: Trials. 2017 Apr 11;18:170. doi: 10.1186/s13063-016-1774-6 (PMC5387231; doi:10.1186/s13063-016-1774-6)
Supplement: Supplementary file 2 — Informed Consent Form. A Multi-center, Double-blind, Double-simulation and Randomized Control Study of Huangkui Capsule for the Treatment of IgA Nephropathy: Informed Consent. (PDF 85 kb) [file 13063_2016_1774_MOESM2_ESM.pdf]

A Multi-center, Double-blind, Double-simulation and Randomized Control Study of Huangkui Capsule for the Treatment of IgA Nephropathy

Informed Consent

Dear patients,

You are invited to participate in the clinical study for the post-marketing re-evaluation of the drug to assess the safety and efficacy of HuangkuiCapsule for the treatment of IgA nephropathy when administered by a large population for a long term and to seek the effective drugs for the IgA nephropathy treatment. The study was listed in the national project of Major New Drugs Innovation and Development (Project No. 2013ZX09104003).

The study will be conducted in 80-100 hospitals nationwide including Chinese PLA General Hospital and approximately 1600 subjects will beincludedon voluntary basis. The study has been reviewed and approved by the Ethics Committee of Chinese PLA General Hospital.

Parts of the contentare defined by the laws and regulation and for securing the rights of the patients involved, the content of the article is reviewed and agreed by the ethnic committee.

Why the study is conducted?

The background: The HuangkuiCapsule is a TCM formulation manufactured by SZYY Group Pharmaceutical Limited with only one TCM component and obtained the market authorization from State Food and Drug Administration in 1999 (MA No. Z19990040, Approval Date: Aug. 13, 1999) with the functions of dampness and heat elimination, detoxification and swelling relieving and indications of damp-heat syndrome induced by chronic nephritis with the symptoms of general edema, backache, proteinuria, hematuria and

yellowish greasy coating of the tongue, etc. The main active ingredient of HuangkuiCapsule is the flavanone. The traditional medicine defines it effective for dampness and heat elimination, detoxification and swelling relieving. The modern medicine discovers its function in decreasing proteinuria, relieving hematuria and regulating the immunity. Sincelaunched more than one decade ago, the Huangkui Capsule has been widely applied for the therapy of primary and secondary chronic kidney disease such as chronic glomerulonephritis, nephrotic syndrome, IgA nephropathy, diabetic nephropathy and hypertensive nephropathy, etc. Numerous literatureshave proved the Huangkui Capsule’s capability to decrease proteinuria, reduce urinary red blood cells, cut down serum creatinine and urea nitrogen, mitigate the tubulo-interstitial damage, and increase plasma albumin as well as improve the kidney condition effectively to finally delay the progress of chronic kidney disease.

IgA nephropathy is the most common primary glomerular disease in China with the proportion of 30%-50%, butthe effective medicine for treatmentis of deficiency. In the earlier stage, we accomplished the clinical trials of Huangkui Capsule for the chronic kidney disease treatment and included 417 subjects diagnosed with primary glomerular disease through renal biopsy in 26 tertiary hospitals. The analysis to the patients with IgA nephropathy displayed the Huangkui Capsule’s effectiveness to reduce the urinary protein of the IgA nephropathy patients. However, two problems exist in the study, that is the insufficient evidence grade of the evidence-based medicine as the open study and inadequate observation duration for 6 months, which may induce the insufficiency of the treatment course.

To further evaluate the safety and efficacy of Huangkui Capsule for the treatment of IgA nephropathy among a large population for a long term, the clinical study will be conducted as one part of the national project of Major New Drugs Innovation and Development (Project No. 2013ZX09104003).

How will the study be operated?

If you are diagnosed with IgA nephropathy clearly and the doctors in charge consider you meet the inclusion criteria of the study, the doctors will arrange you participate in the study on voluntary basis.

During your hospitalization and the follow-ups lasting one year after discharge, the doctor will collect your general information, illness history, medications for treatment, adverse events and various examination results for better evaluatingyour disease condition. When issues occur, the doctors will handle them properly and timely.

What should I do in the study?

The study will last one year and six visits will be included in total. You should return visit following the schedule under the instruction of your doctor. In addition to the routine treatment, the doctor will seek your cooperation for the collection of treatment data and copies of your examinations reports.

During visit 1, the doctor will make an evaluation to you according to the inclusion and exclusion criteria, and meanwhile collect your general information, illness history and labs examination results.

The visit 2 will be the 1 month ±7 days after your treatment initiation.

The visit 3 will be the 3 months ±7 day after your treatment initiation.

The visit 4 will be the 6 months ±7 days after your treatment initiation.

The visit 5 will be the 9 months ±7 days after your treatment initiation.

The visit 6 will be the 12 months ±7 days after your treatment initiation.

During the visits 2～6, you should return visit the hospital for the routine clinical examinations. The return visits are very important to you because the doctor will determine whether your treatment is effective or whether your disease condition changes. During the return visit, you should tell the doctors about your treatment situation honestly. The doctor will collect your disease history and examination results, as well as leave your blood and

urine for further labs examination. The doctors will specify and give you a guidance on the detailed information and procedures.

If any adverse event occurs, please contact your doctor in charge immediately.

How will the study affect my life?

During the study duration, neither your medical treatment nor life will be affected except you should cooperate to follow ups and provide required data and information (the routine examination of normal clinical fellow up treatment for IgA nephropathy)

What risks and adverse events may exist if I am involved in the study?

The study will follow the Good Clinical Practice, the World Medical Association Declaration of Helsinki, and International Ethical Guidelines for Biomedical Research Involving Human developed by Council for International Organizations of Medical Sciences strictly to secure the dignity, safety and rights of the subjects. The treatment protocol adopted in the study is the mature therapy protocol for IgA nephropathy treatment clinically and the medicine applied is the drug that has been approved for marketing by China Food and Drug Administration, which will not bring about extra risks and adverse reactions to you.

Tell your families and your intimate friends that you are participating in one clinical trial. If they have any doubt about the study, please give them the contact information of your doctors.

How the study will benefit me?

You will be effectively treated through the trial. You will obtain the observing medicine free of charge, which will reduce your medications cost by more than 4000 RMB per year. During the study term, you will get the detailed treatment instruction of the doctors, which means

your disease progress will be monitored and your treatment will be guided in a better way. You will acquire part of the lab examinations for free. During the study, your doctor will inform you timely of the new and vital information about your treatment, if any.

How much will I be paid in the study?

The study is a clinical trial under the financial assistance of the national project of Major New Drugs Innovation and Development, and you will not be paid personally.

What if I am harmed during the study?

The treatment protocol adopted by the clinical study is one mature protocol for the IgA nephropathy treatment in clinical, and the treatment drugs has been approved for marketing by China Food and Drug Administration, which will not bring any extra harm to your disease treatment. As any other medicine, some adverse reactions such as allergy and abdominal distention may occur to a small amount of patients. If you are female, your will be forbidden taking part in the study during your pregnancy and lactation. Pregnancy is forbidden during the study. If you have an accidental pregnancy, please let your doctors know immediately so as to track the drugs' effect on your pregnancy. Any discomfort occurs or the disease condition changes, whether related to the study or not, please inform your doctors and researcher as soon as possible so that timely guidance from them could be given.

The doctors and the sponsorSZYY Group Pharmaceutical Limited will spare no efforts to avoid the study induced harm to you. The Medical Experts Committee will identify whether the adverse events happen in the study is related to the test drug. If the adverse reactions are caused by the test drug and are harmful to your health, we will provide timely treatmentto you according to the applicable laws and regulations.

Will my personal information be confidential?

Your medical record will be preserved in the hospitals and the researcher, authority department of the study and the Ethnic Committee will be allowed to review them. Any public report on the study result will not disclose your identity, and your privacy about the personal medical information will be well protected to the full extent permitted by law.

Your personal and medical information will be kept confidential to the public and preserved in the safe and reliable place. Your personal information (such as your name and address) can be referred to at any time by yourself if requested, and amended if necessary.

The Ethnic Committee of Chinese PLA General Hospital has considered the study safe and comply with the medical ethnic standards and has approved the clinical study. If you agree to participate in the study, you will permit the sponsor or its representatives, ethnic committee or the authorities inspecting or supervising your medical records or health information. The data or information collected in the study duration may be delivered to the corresponding authority or published, but none of your identity information will be disclosed. Your signing on the informed consent means your personal or medical information may be applied in the situations described above.

Do I have to participate in the study?

The study is on voluntary basis and you reserve the right to consult any question about the study and the right to withdraw from the studyat any time. Once you withdraw the study for whatever, you may choose other treatment under the guidance of the doctors. Your participation or refusal of the study will affect neither the patient-doctor relationship nor your future treatment.

If you decide to withdraw the study, please inform your doctors in advance. For securing your safety, you may be asked for some examinations, which is beneficial for protecting your health.

The consent statement of the subjects:

I have read the above introduction to the study and got a thorough understanding of the potential risks and benefits. I agree to participate in the clinical study described in the text and get known I will get a copy of counterpart of the informed consent. I will be permitted to withdraw from the study without regardless the reasons at any time after I participate the observation, which will not affect my treatment in the future.

Please sign your name on the informed consent of the last page if you understand and agree to take part in the observation.

Signature Page of the Informed Consent

Name of study: A Multi-center, Double-blind, Double-simulation and Randomized Control Study on Huangkui Capsule for the Treatment of IgA Nephropathy.

Sponsor: SZYY Group Pharmaceutical Limited

Code of the Major New Drugs Innovation and Development: 2013ZX09104003

Manufacturing license No. of Huangkui capsule: Z19990040

Ethical approval No. of Chinese PLA General Hospital: S2014-039-01

Statement of consent:

I have read the above introduction to the study, got the opportunity to discuss with the doctors about the study and received satisfactory replies to all the questions I brought up.

I have known all the potential risks and benefits of the study I will participate in. I know the participation is on the voluntary basis and make sure I have enough time for thinking about it, and I understand:

- I am able to consult the doctors for more information at any time;
- I am able to withdraw from the study at any time without being discriminated or retaliated or inducing any effect on my medical benefits or rights.

I am also clear that if I drop out of the study especially due to the medication, it will be very beneficial to myself and the study to tell the doctor my disease conditionand complete the physical examination and physio-chemical examinations.

If I need other medicine treatment due to the disease changes, I will ask for the doctor's suggestion in advance or tell the doctor honestly afterwards.

I will get one signed and dated counterpart of the informed consent. At the same time, I

permit the China Food and Drug Administration, Ethnic Committee and relevant studies personnel reviewing my medical record.

Above all, I decide to take part in this study.

Signature of the subject:Date: (YYYYMMDD)

Phone: Mobile:

I make sure the detailed information of the study including the rights and potential risks and benefits of the patients have been explained to and the signed and dated counterpart have been given to the patients.

Signature of the doctor:Date:(YYYYMMDD)

Office No. of the doctor:Mobile:
